# The Vacuum Assisted Negative Pressure Isolation Hood (VANISH) System: Novel Application of the Stryker Neptune™ Suction Machine to Create COVID-19 Negative Pressure Isolation Environments

**DOI:** 10.7759/cureus.8126

**Published:** 2020-05-14

**Authors:** David Convissar, Connie Y Chang, Wonjae E Choi, Marvin G Chang, Edward A Bittner

**Affiliations:** 1 Anesthesiology and Critical Care, Massachusetts General Hospital, Boston, USA; 2 Anesthesiology, Mercy General Hospital, Sacramento, USA; 3 Anesthesia, Critical Care, and Pain Medicine, Massachusetts General Hospital, Boston, USA

**Keywords:** coronavirus disease (covid-19), negative pressure rooms, negative pressure hood, negative pressure tent, health care worker safety, covid-19, wall suction, stryker neptune, vanish system, vacuum assisted negative pressure isolation hood

## Abstract

Coronavirus disease 2019 (COVID-19) may remain viable in the air for up to three hours, placing health care workers in close proximity to aerosolizing procedures particularly at high risk for infection. This combined with the drastic shortage of negative pressure rooms hospitals worldwide has led to the rapid innovation of novel biohazard isolation hoods, which can be adapted to create negative pressure isolation environments around the patient's airway using the hospital wall suction, which carries many limitations, including weaker suction capabilities, single patient use, and immobility. Here, we report our Vacuum Assisted Negative Pressure Isolation Hood (VANISH) system that uses a mobile and readily available in most hospital operating rooms Stryker Neptune™ (Stryker Corporation, Kalamazoo, Michigan) high-powered suction system to more effectively create a negative pressure biohazard isolation environment. VANISH has been utilized regularly in an anesthesia practice of 30+ providers and, to date, there have been no documented COVID-19 infections.

## Introduction

The severe acute respiratory syndrome coronavirus 2 (SARS-COV-2) virus responsible for COVID-19 is transmissible via aerosolization and may be viable in the air for at least three hours [1-2]. The increased need for aerosolizing procedures, such as endotracheal intubation, extubation, tracheostomy, cricothyrotomy, endoscopy, bronchoscopy, transesophageal echo (TEE), high flow nasal cannula (HFNC), and noninvasive ventilation (NIV), such as bilevel positive airway pressure (BiPAP), continuous positive airway pressure (CPAP), and bronchoscopy, combined with the shortage of negative pressure rooms has led to the rapid innovation of novel biohazard isolation hoods adapted to use wall suction to create a negative pressure environment for patients with COVID-19 [3-7]. While effective, these wall suctions systems have certain limitations, such as immobility, single patient use, and weaker suction capabilities. Here, we report the first use of the Stryker Neptune™ (Stryker Corporation, Kalamazoo, Michigan) high-powered suction systems that are mobile-ready and available in most hospital operating rooms to more effectively create negative pressure biohazard isolation hoods so as to minimize risk to healthcare workers, performing aerosolization procedures on COVID-19 confirmed and suspected patients. This novel negative pressure isolation biohazard hood has been utilized regularly in an anesthesia practice of 30+ providers and, to date, there have been no documented COVID-19 infections.

## Technical report

The Vacuum Assisted Negative pressure ISolation Hood (VANISH) system

The first report of an isolation biohazard hood for use by healthcare providers to protect against SARS-COV-2 aerosolization procedures was developed by Dr. Justin Henneman at New York-Presbyterian [4]. His simple design consisted of Polyvinyl chloride (PVC) piping as a frame, which was then covered by a clear plastic drape.

**Figure 1:**
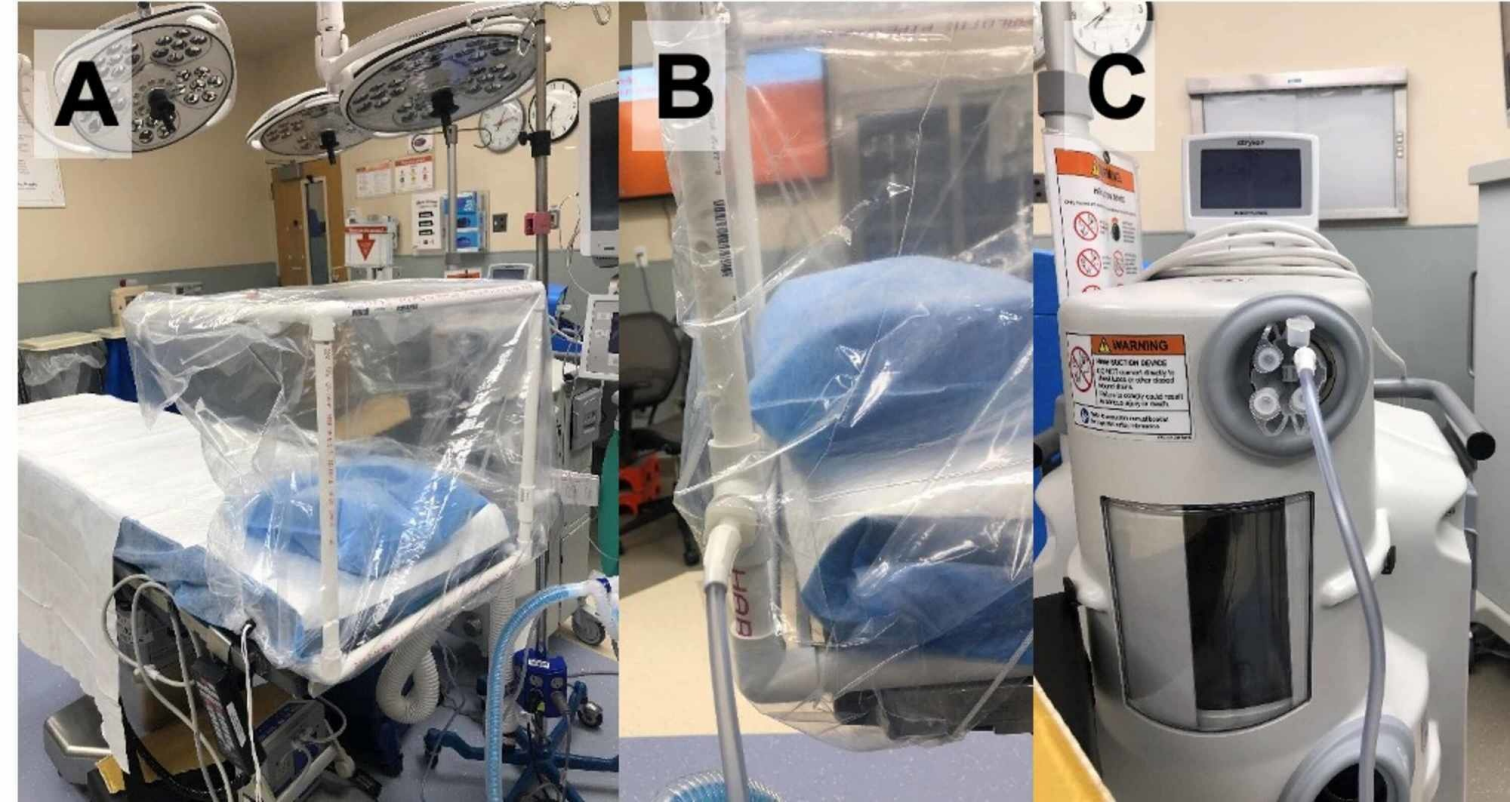
Negative Pressure Isolation Biohazard Hood Panel A shows a clear plastic bag with drawstrings is placed over the hood. Panels B and C show suction tubing connected to the proximal suction port of the hood and Neptune Stryker, respectively. Stryker Neptune™: Stryker Corporation, Kalamazoo, Michigan

**Figure 2:**
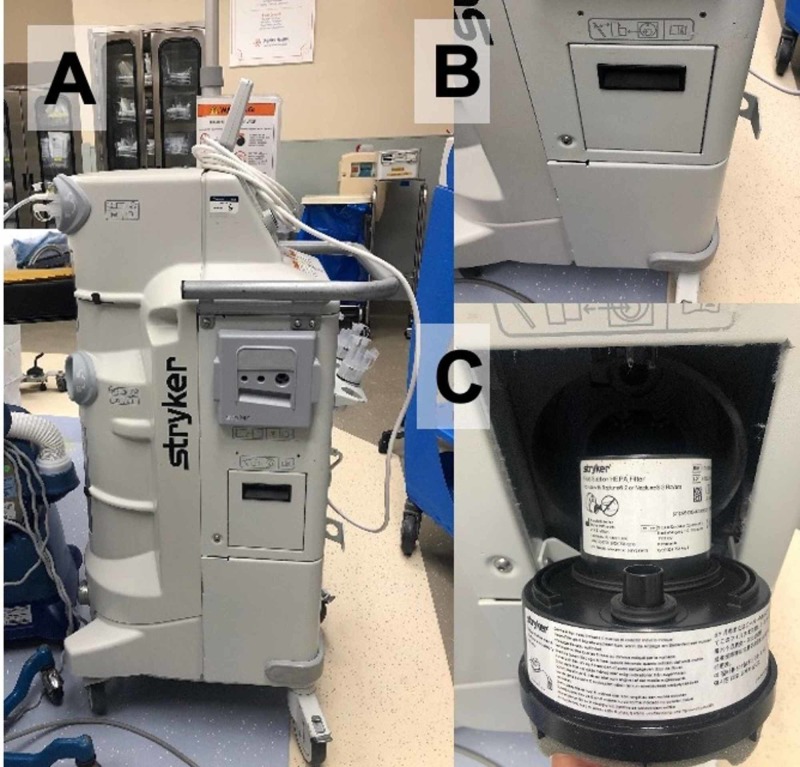
Stryker Neptune Suction System With a HEPA Filter Panel A shows the side profile of the Stryker Neptune suction system. Panels B and C show the enclosure housing the HEPA filter closed and opened, respectively. HEPA: high-efficiency particulate air; Stryker Neptune™: Stryker Corporation, Kalamazoo, Michigan

Our novel design expands on Dr. Henneman’s basic design by using high-pressure suction to create a negative pressure environment within the biohazard hood. As a result, aerosolized particles are continuously evacuated, protecting the clinician who is performing an airway-related procedure at the head of the bed. Our clear plastic bag is used to enclose the PCV frame (Figure 1) and high-pressure suction is applied via a Stryker Neptune™ with a pre-installed high-efficiency particulate air (HEPA) filter (Figure 2) to create a negative pressure environment within the enclosure.

**Figure 3:**
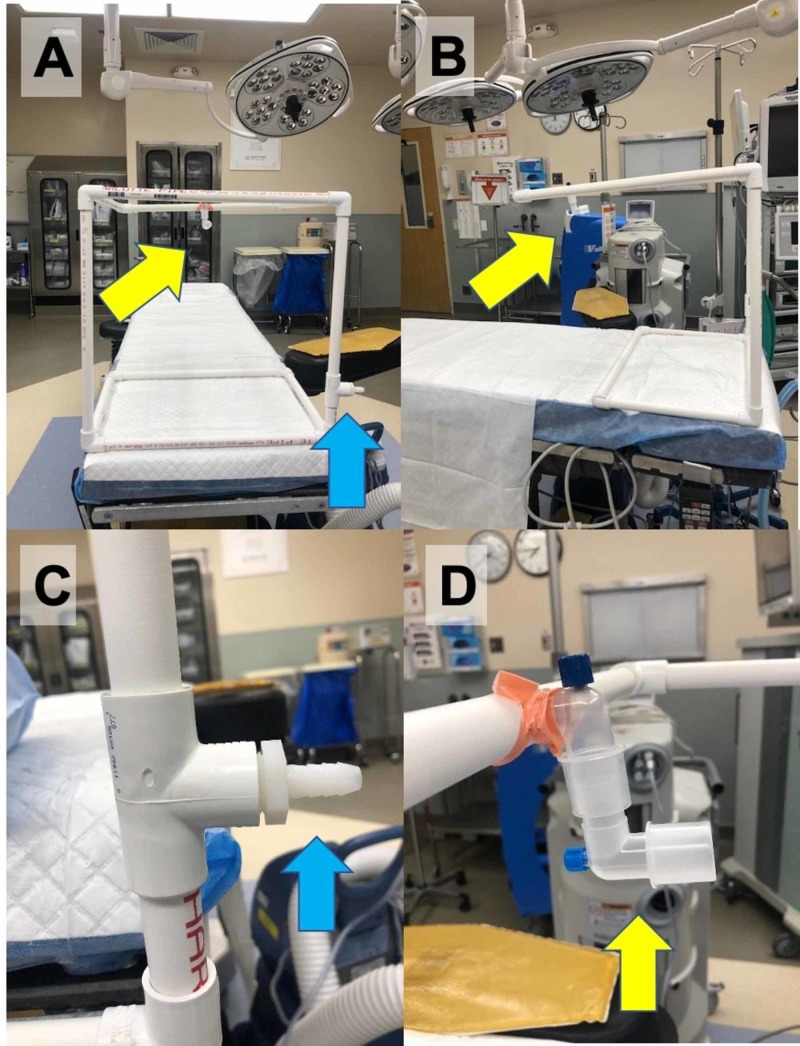
PVC Isolation Biohazard Hood Frame With Suction Ports Panels A and B show the lateral and head-on views of the hood, respectively. The proximal suction port (blue arrow) attaches to the Stryker Neptune to create a negative pressure within the PVC tubing to pull aerosolized particles towards the distal suction port (yellow arrow) away from the head of the patient. Panels C and D show closeup images of the proximal and distal suction ports, respectively. PVC: polyvinyl chloride; high-efficiency particulate air; Stryker Neptune™: Stryker Corporation, Kalamazoo, Michigan

Figure 3 shows our design of a similar PVC isolation biohazard hood frame that was adapted to include suction ports to create a negative pressure environment within the isolation biohazard hood.

## Discussion

Technological principles and viability

A negative pressure room is created by removing air from the room at a rate than it is being replaced. This results in airflow into the room from outside (pressurized to non-pressurized flow) via a vacuum effect. A ventilation system in place then filters the air and then exhausts the air outside using a vacuum effect [8].

Conventional wall suction in the operating room after being connected to a vacuum regulator generates a maximum negative pressure of -200 mmHg resulting in a flow of 80.2 liters per minute of airflow. After all tubing, canisters, regulators, and a Yankauer suction tip are attached, the effective flow rate is reduced to 47.6 liters per minute of air movement [9]. In contrast, the Stryker Neptune™ generates significantly greater suction pressure, reaching maximum negative pressures of 520 mmHg per suction canister [10]. Thus, the air exchange rate and the efficiency of aerosolized particle removal is much greater with a Stryker Neptune™ suction as compared to the wall suction. Furthermore, a single Stryker Neptune™ suction system may be used to generate sufficient negative pressure for multiple patients.

Advantages of using the VANISH system

An additional advantage of using the Stryker Neptune™ is that it is designed to allow a HEPA filter to be attached to the device and this filter does not need to be replaced with each new patient using it. Because of the increasingly limited supply of HEPA filters, this could be key to the utilization of these hoods. Furthermore, if the in-line Stryker Neptune™ HEPA filter is not available, a standard HEPA filter can easily be attached to the distal suction port of the PVC isolation biohazard hood.

The substantially greater amount of suction created by the Stryker Neptune™ can also be divided between multiple suction canisters that are part of the system, thereby allowing use for multiple patients undergoing aerosolizing procedures independently and simultaneously. In comparison, the standard hospital wall suction is only ideal for a single patient.

The mobile nature of the Stryker Neptune™ is ideal to facilitate the use of negative pressure hoods in arenas where wall suction is not consistently available such as in emergency room hallways. The ability to transport the Stryker Neptune™ device from room to room to create negative pressure environments also removes the need to move patients to scarcely available negative pressure rooms in the hospital.

Because the hood is made of clear plastic, practitioners can cut slits in the sides in order to interact with patients. The holes are then resealable with Tegaderm. In this way, interventions such as adjusting a mask or extubating or intubating a patient can be performed and the hood can then be safely re-sealed.

The VANISH system can be used for a variety of aerosolizing procedures such as BiPAP, CPAP HFNC, endotracheal intubation, extubation, bronchoscopy, endoscopy, TEE, cricothyrotomy, and tracheostomy. Due to the space limitation within the VANISH system and visibility issues secondary to the plastic drape, it may be difficult to perform more complicated surgical procedures within the hood.

Real-world application of the VANISH system

A 30-physician private practice anesthesia group in Sacramento, California, has used the VANISH system routinely for aerosolizing procedures for an over one-month period. Even with regular use, the practice has had no documented infections to date despite practicing in a community in which the first case of community-acquired COVID-19 pneumonia was reported in the US [11]. It is important to note that anesthesiologists are among the at-risk health care workers for acquiring COVID-19 infection given the high risk for aerosolization during intubation and extubation.

Limitations of using the VANISH system

The availability of a Stryker Neptune™ suction system is the primary limitation in the routine implementation of this system. However, many hospitals use these Stryker Neptune™ suction systems for operating room (OR) procedures and, therefore, have them readily available. If the care of multiple patients required the use of the biohazard hood or procedures requiring its use for a prolonged duration (e.g., CPAP) then the Styker system would be unavailable to the operating room during that time period.

Furthermore, the incorrect use of the Stryker Neptune™ system could have devastating consequences. A case in which the Stryker Neptune™ was inappropriately attached to a patient’s chest tube after thoracotomy has been reported, which resulted in massive hemorrhage and death [12]. In another event, the device was connected to a chest tube during a pneumonectomy that resulted in a pronounced shift of the mediastinum and tearing of the aorta [13]. Thus, appropriate education is essential for the use of the Stryker Neptune™ as a suction to avoid and minimize such complications.

We did not perform formal quantitative testing of our negative pressure isolation biohazard hood prior to use. Its utility is demonstrated by the anesthesiologists who safely and effectively use the technology as part of their everyday clinical practice.

## Conclusions

During the COVID-19 pandemic, the increased need for aerosolizing procedures as part of patient care along with limitations in the availability of negative pressure rooms has resulted in innovation to ensure the safety of medical practitioners. Here, we have demonstrated the functional application of a Stryker Neptune™ suction machine to create mobile negative pressure isolation environments to facilitate aerosolizing procedures.
